# Digenic Inheritance of Shortened Repeat Units of the D4Z4 Region and a Loss-of-Function Variant in *SMCHD1* in a Family With FSHD

**DOI:** 10.3389/fneur.2018.01027

**Published:** 2018-11-28

**Authors:** Raffaella Cascella, Claudia Strafella, Valerio Caputo, Rosaria Maria Galota, Valeria Errichiello, Marianna Scutifero, Roberta Petillo, Gian Luca Marella, Mauro Arcangeli, Luca Colantoni, Stefania Zampatti, Enzo Ricci, Giancarlo Deidda, Luisa Politano, Emiliano Giardina

**Affiliations:** ^1^Molecular Genetics Laboratory UILDM, Santa Lucia Foundation, Rome, Italy; ^2^Department of Chemical-Toxicological and Pharmacological Evaluation of Drugs, Catholic University Our Lady of Good Counsel, Tirana, Albania; ^3^Department of Biomedicine and Prevention Tor Vergata University, Rome, Italy; ^4^Emotest Laboratory, Pozzuoli, Italy; ^5^Department of Experimental Medicine, Cardiomyology and Medical Genetics, University of Campania Luigi Vanvitelli, Naples, Italy; ^6^Department of Experimental Medicine and Surgery, University of Rome Tor Vergata, Rome, Italy; ^7^Institute of Neurology, Catholic University of the Sacred Heart, Rome, Italy; ^8^Institute of Cell Biology and Neurobiology, National Research Council of Italy, Monterotondo, Rome, Italy

**Keywords:** facioscapulohumeral muscular dystrophy, *DUX4* gene, *SMCHD1* gene, methylation analysis, neuromuscular symptoms, genetic counseling, familial investigation

## Abstract

Facioscapulohumeral muscular dystrophy (FSHD) is a neuromuscular disorder which is typically transmitted by an autosomal dominant pattern, although reduced penetrance and sporadic cases caused by *de novo* mutations, are often observed. FSHD may be caused by a contraction of a repetitive element, located on chromosome 4 (4q35). This locus is named *D4Z4* and consists of 11 to more than 100 repeated units (RU). The *D4Z4* is normally hypermethylated and the genes located on this locus are silenced. In case of FSHD, the *D4Z4* region is characterized by 1–10 repeats and results in the region being hypomethylated. However, 5% of FSHD cases do not carry the short allele of *D4Z4* region. To date, two forms of FSHD (FSHD1 and FSHD2) are known. FSHD2 is usually observed in patients without the *D4Z4* fragment contraction and carrying variants in *SMCHD1* (18p11.32) gene. We report the case of a young adult patient who shows severe symptoms of FSHD. Preliminary genetic analysis did not clarify the phenotype, therefore we decided to study the family members by genetic and epigenetic approaches. The analysis of *D4Z4* fragment resulted to be 8 RU in the affected proband and in his father; 26 RU in the mother and 25 RU in the maternal uncle. *SMCHD1* analysis revealed a heterozygous variation within the exon 41. The variant was detected in the proband, her mother and the uncle. Furthermore, epigenetic analysis of CpG6 methylation regions showed significant hypomethylation in the affected patient (54%) and in the mother (56%), in contrast to the father (88%) and the uncle (81%) carrying higher methylation levels. The analysis of DR1 methylation levels reported hypomethylation for the proband (19%), the mother (11%), and the uncle (16%). The father showed normal DR1 methylation levels (>30%). Given these results, the combined inheritance of *SMCHD1* variant and the short fragment might explain the severe FSHD phenotype displayed by the proband. On this subject, *SMCHD1* analysis should be promoted in a larger number of patients, even in presence of *D4Z4* contractions, to facilitate the genotype-phenotype correlation as well as, to enable a more precise diagnosis and prognosis of the disease.

## Introduction

Facioscapulohumeral muscular dystrophy (FSHD; OMIM #158900 and #158901) is one of the most common adult form of myopathy, with an overall incidence of 1 in 8,300 individuals. FSHD is described as a heterogeneous disorder characterized by progressive weakness of the shoulder girdle, facial, humeral, and, at later stages, of the trunk muscles (1). The onset of disease can occur at any age, from the infancy to the advanced age. In some cases, anticipation of disease symptoms has also been described (2). Approximately 10% of cases display the first symptoms before age 20 (infantile or early onset forms, namely iFSHD), reporting a severe muscle symptomatology with concomitant hearing loss (often bilateral), eye problems (retinal telangiectasias), and seizures (1, 3).

FSHD is generally transmitted by an autosomal dominant pattern, although reduced penetrance and sporadic cases caused by *de novo* mutations are often observed (1, 4). FSHD is associated with a contraction of a repetitive element, located on a subtelomeric region of chromosome 4 (4q35). This locus is named *D4Z4* and consists of 11 to more than 100 repeated units (RU), extending for 3.300 DNA base pairs (3.3 kb) in length. In normal conditions, *D4Z4* is hypermethylated and the genes included within this region are silenced. One of these genes is *DUX4*, which is actively expressed during the early development and in the testes of adult males, while it is turned-off in most adult cells and tissues (4). In presence of FSHD, the *D4Z4* repeats are found to be shortened up to 1–10 repeats (short-allele) causing thereby a significant hypomethylation of the distal *D4Z4* repeats (which are normally hypermethylated). This condition results in a de-repression of *DUX4* in cells and tissues, whose activation causes myofiber death, defects of myogenesis regeneration, and a higher sensitivity to oxidative stress (1). Furthermore, *DUX4* is located in the proximity of a DNA regulatory region named pLAM sequence, that is crucial for the DUX4 protein expression and functioning. The active form of pLAM sequence is called 4qA or “*permissive*” allele, while the inactive sequence is named 4qB or “*non-permissive*” allele. Interestingly, 4qA allele is also present on chromosome 10q26, although it is not associated with FSHD. A deeper characterization of 4q35 region led to the identification of different haplotypes of the 4qter, including 4qA161, 4A159, and 4A168 (5). In particular, 4qA161 is normally associated with FSHD clinical phenotype in combination with the short allele. However, it is important to remark that the presence of 4qA allele and the short allele in *D4Z4* region do not necessarily cause the onset of FSHD. In fact, a small fraction of FSHD cases (5%) do not carry the short allele in *D4Z4* region, which can be present in healthy people as well. Such a variable penetrance is peculiar of FSHD and it is generally associated with different genetic, epigenetic, and environmental factors (1, 6–8).

Besides the classical form of FSHD (FSHD1), another form of disease (FSHD2, OMIM #58901) is also known, although it is less common than FSHD1. FSHD2 clinical phenotype is usually observed in patients who do not present the *D4Z4* fragment contraction (5, 9–14) and it is associated with variants in *SMCHD1* (18p11.32) gene. The homonymous protein (SMCHD1) modulates the hypermethylation of *D4Z4* and contributes to the maintenance of the chromosome structures. *SMCHD1* variants alter the encoded protein, inducing thereby the hypomethylation of *D4Z4* region and, consequently, the expression of DUX4 protein (15). Given its impact on DUX4 expression, variants of *SMCHD1* have also been shown to act as disease modifiers and can affect the severity of FSHD1. FSHD1 and FSHD2 share the same clinical features and present DUX4 expression. However, the lack of a precise genotype-phenotype correlation in many cases explain the need for a more comprehensive genetic analysis of both FSHD1 and FSHD2 genes at a familial level whenever possible.

Here, we report the case of a young adult patient showing symptoms consistent with FSHD. Preliminary genetic analysis did not clarify the phenotype, therefore we decided to study the family members by genetic and epigenetic approaches.

## Case presentation

### Clinical characterization of patients

The clinical assessment of patients was performed by using the Comprehensive Clinical Evaluation Form (CCEF), which has been recently set up by the Italian Clinical Network for FSHD to assign each patient to the specific clinical category. In particular, subjects presenting facial and scapular girdle muscle weakness are classified as category A; patients with only facial or scapular weakness fall into category B. Subjects presenting symptoms of category A together with additional atypical features, are classified as Category D1; those who present clinical myopathic features not consistent with FSHD are referred as category D2, while apparently healthy individuals are classified as category C (16).

Patients belong to a two-generation family originating from the province of Naples. At the medical visit, the proband was 18–25 years old and complained scapular weakness associated with a mild increase (2x) in CK values. Muscular examination showed mild hypotrophy of periscapular muscles with bilateral scapular winging and difficulties in raising the arms above the shoulders, hypotrophy of pectoralis and triceps muscles. At the age of 20–22, he underwent surgical fixation of shoulders and his muscle condition remained stable for more than 15 years.

Successively, the maternal uncle reported some difficulties in walking and prevalent weakness at the upper limbs. In addition, even the mother of the proband started to experience moderate difficulties in walking and a severe weakness of the trunk and limb muscles, in combination with a mild increase of CK values. The father of the proband did not show any symptoms suggestive of a FSHD phenotype. The study was approved by the ethics committee of Santa Lucia Foundation and was performed according to the Declaration of Helsinki. All participants provided signed informed consent and, in this regard, all the recruited patients also provided the consent for publication of this case report.

### Laboratory investigations and diagnostic tests

The first step of analysis was performed by Southern blot and Pulsed-Field Gel Electrophoresis (PFGE). To this purpose, DNA was extracted from lymphocytes according to standard procedures. Restriction endonuclease digestion of DNA was performed on agarose plugs with specific restriction enzyme, such as EcoRI, EcoRI/BlnI, and XapI. Digested DNA was separated by PFGE in 1% agarose gel for 11 h. The *D4Z4* RU were evaluated by Southern blotting utilizing 7 mg of EcoRI-, EcoRI/BlnI-, and XapI-digested genomic DNA. The assay was hybridized with p13E-11 probe. The results were confirmed by Linear Gel Electrophoresis (LGE) on 0.4% agarose gel for 45–48 h. In addition, 4qA and 4qB alleles were subjected to digestion with HindIII and run on PFGE. Subsequently, the DNA was analyzed by Southern blot hybridization with radio-labeled 4qB and 4qA probes, according to standard procedures. The presence of 4qA and 4qB alleles was compared to the results obtained by the digestion with EcoRI enzyme.

Afterwards, patients were subjected to a second step analysis, in order to evaluate the whole genetic architecture of *SMCHD1* by Next-Generation Sequencing (NGS). Therefore, genomic DNA was re-extracted from 400 μL of peripheral blood using MagPurix Blood DNA Extraction Kit and MagPurix Automatic Extraction System according to the manufacturer's instructions. The extracted DNA was quantified by DeNovix Spectrophotometer. *SMCHD1* gene was sequenced using Ion Torrent S5 and Ion Ampliseq LGMD Panel, designed by Ion Ampliseq Designer (Thermo Fisher Scientific). The construction of the library was performed by Ion AmpliSeq™ Library Kits 2.0 and the library quality was evaluated by Qubit® 2.0 Fluorometer. The enrichment procedures were performed by Ion Chef System (Thermo Fisher Scientific). The results were analyzed using Ion Reporter 5.2 (Thermo Fisher Scientific), Integrated Genome Viewer (IGV) and using NM_015295.2 as cDNA reference sequence. The interpretation of genetic variants was conducted by Human Gene Mutation Database (HGMD), Leiden Open Variation Database (LOVD), ClinVar, ExaC, GnomAD, and 1000 Genome Browser. The functional effect of the detected variants was evaluated by bioinformatic predictive tools such as SIFT, Poly-phen 2, Provean, Mutation Taster, and SMART. The genetic variants were confirmed by direct sequencing (BigDye Terminator v3.1, BigDyeX Terminator and ABI3130xl, Applied Biosystems).

The samples were subsequently utilized for SSLP (Simple Sequence Length Polymorphism) analysis, in order to assess the epigenetic profile of the proband and his family members. SSLP assay was performed as described in Lemmers et al., (17). Genomic DNA was amplified using forward primer 5′- HEX-GGTGGAGTTCTGGTTTCAGC-3′ and reverse primer 5′-CCTGTGCTTCAGAGGCATTTG-3′. Size analysis was performed using an ABI Prism 3,100 genetic analyzer. Presence of B (4qB) alleles was performed by PCR with B-specific primers on bisulfite converted DNA as previously described (18). Two hundred nanogram of genomic DNA from each subject were converted using EZ DNA Methylation-Direct Kit (Zymo Research) following manufacturer's instruction. Two different regions were amplified: (1) the region distal to the D4Z4 array (pLAM) containing the DUX4-PAS (4qA); (2) the region within each D4Z4 unit both on 4q and 10q chromosomes (DR1). Primers were designed with MethPrimer software. PAS-specific PCR and B-specific PCR were performed as previously described (18). The DUX4 PAS-specific bisulfite product (235 bp) contains 10 CpG and DR1 product encloses 30 CpGs (19). Sequencing of Bisulfite (BSS) products was performed using BigDye terminator v3.1 Cycle Sequencing Kit. Samples were subjected to capillary electrophoresis on the ABI Prism 3100 (Applied Biosystem) and subsequently analyzed with the ABI prism DNA sequencing software and Gene Mapper software 4.0 (18).

## Results

**Figure 1:**
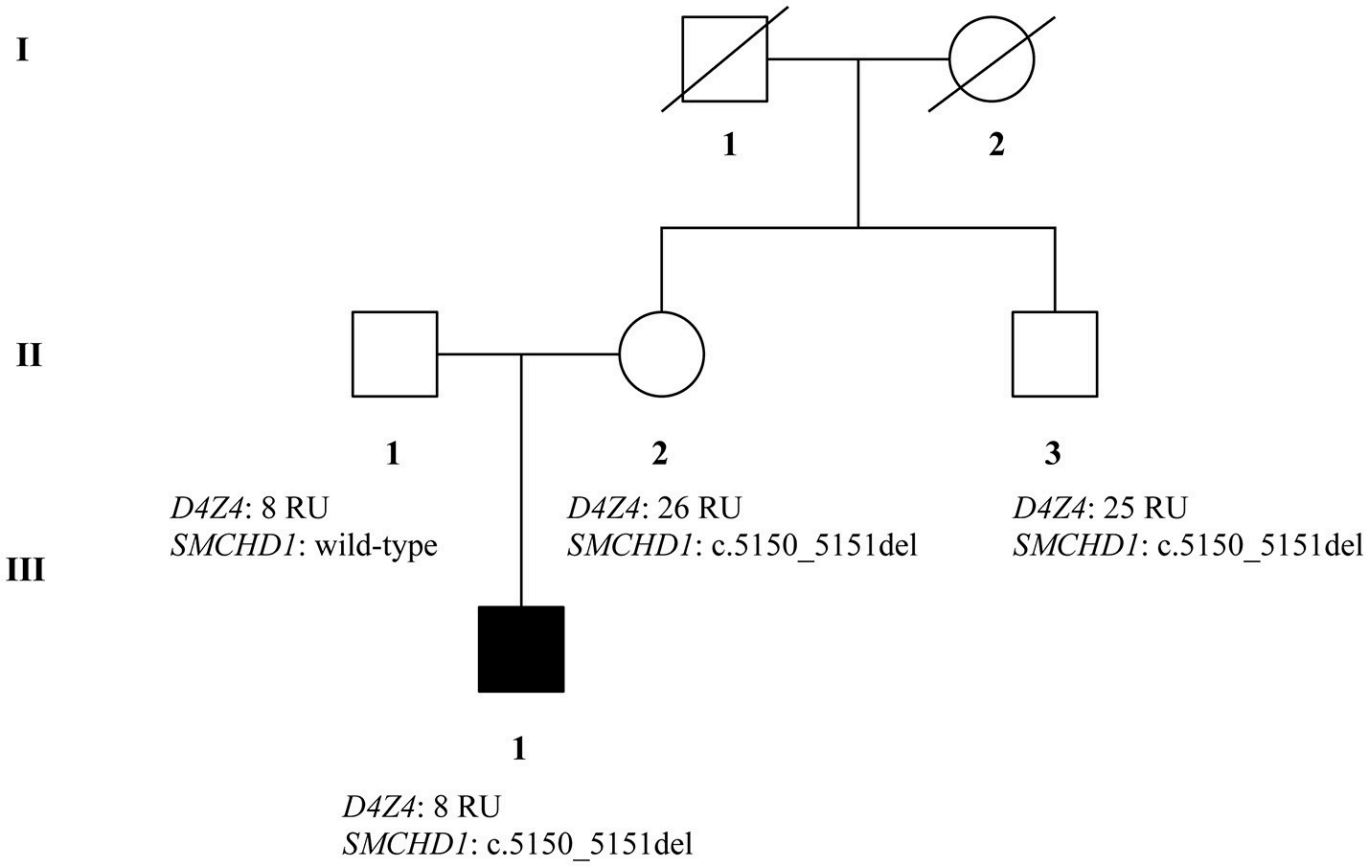
Genetic pedigree of analyzed family with *D4Z4*and *SMCHD1* genotypes.

**Figure 2:**
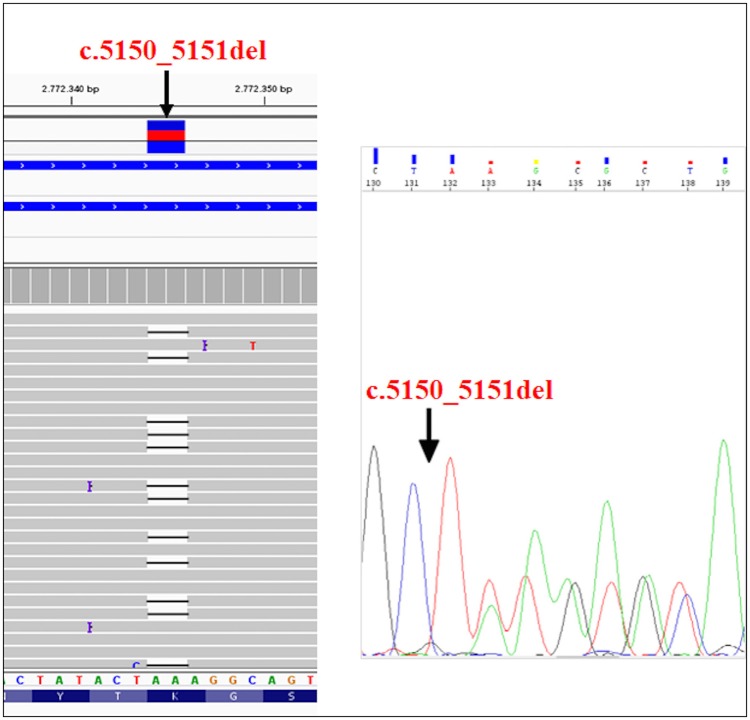
NGS analysis of *SMCHD1*gene and subsequent confirmation of the variant (c.5150_5151del) by traditional sequencing.

The recruited subjects were characterized by genetic and epigenetic analysis. The analysis with p13E-11 probe revealed a *D4Z4* fragment of 8 RU (36 Kb) in the affected proband and in his father. A 26 RU fragment (95 Kb) was reported in the mother, while the maternal uncle presented a 25 RU (93 Kb) fragment. All subjects resulted to be carriers of the 4qA allele. However, the presence of the 8 units fragment and of the 4qA allele in the index case did not fully explain his clinical phenotype. To this purpose, the genetic investigation has been extended to the *SMCHD1* gene in the proband and its relatives (Figure 1). NGS analysis, performed on the proband, revealed a heterozygous variation within the exon 41 of *SMCHD1*. In particular, the detected variant is a rare deletion variant, namely c.5150_5151del (p.Lys1717fs). The variant was confirmed by traditional sequencing (Figure 2) within the proband and the family members. In addition, the variant has not been detected in 100 control subjects, suggesting that it is likely to be pathogenetic.

The identified variant was not present in most of the online variants annotation database and prediction tools, except for Mutation Taster, which described it as a disease-causing variant. Mutation Taster results suggested that the variant creates a frameshift and a PTC (Premature Termination Codon) leading to nonsense-mediated mRNA decay (NMD). In addition, the analysis of variant by SMART prediction tool revealed that the truncated protein may loss the SMC hinge domain that is essential for the functioning and the activity of SMCHD1 (15, 20, 21). According to the criteria established by the American College of Medical Genetics (ACMG) Standards and Guidelines, it is classified as a 5th class variant, meaning that it is pathogenetic for the disease. In particular, this variant can be classified as PVS1 because it is a null variant that is potentially able to cause loss of function in *SMCHD1*; PM2 since it is absent in Exac, GnomAD, and 1000 Genome Browser databases; PM4 as the variant creates a stop codon altering the protein length and PP1 because of the evidence of segregation in the affected family members (22).

Furthermore, the methylation analysis has been performed on the region distal to the *D4Z4* using a PAS specific assay, which allows the evaluation of the methylation pattern of permissive alleles. Normally, the methylation level at CpG6 is below 72% in FSHD1 and FSHD2 (18). Significant CpG6 methylation levels were reported in the affected proband (III:1) and in the mother (II:2) which presented 54 and 56% methylation level, respectively. The healthy father (II:1) and the maternal uncle (II:3) showed higher methylation values (88 and 81%, respectively) (Table 1). Since general hypomethylation of *D4Z4* units has been associated with FSHD2 (1, 19), we also investigated the DR1 region. This analysis measures methylation as the mean methylation of all DR1 regions independently of their chromosomal origin (chromosome 4 or chromosome 10) and it is generally below 30% in subjects with FSHD2 (19). In this case, we observed reduced methylation levels for subjects III:1, II:2, and II:3 who displayed 19, 11, and 16% values, respectively.

**Table 1:** Methylation levels of PAS and DR1 regions in the analyzed family.

**Patients**	**CpG6 Methylation levels (in%)**	**DR1 Methylation levels (in%)**
II:1	88	>30
II:2	56	11
II:3	81	16
III:1	54	19

## Conclusions

The molecular features of FSHD have been investigated in a family presenting mild and severe neuromuscular symptoms. Genetic analysis has been performed on the proband suffering from severe FSHD, the healthy father, the mother and the maternal uncle, who both showed clear clinical features of mild FSHD. The assessment of *D4Z4* fragment length was performed on the collected samples, identifying a short allele (8 RU fragment) in the proband and his healthy father, while the mother and the maternal uncle showed normal *D4Z4* repeats. However, *D4Z4* contraction was not enough to explain the presence of the disease and the severe phenotype in the proband. Therefore, the genetic analysis has been extended to the *SMCHD1* gene, which is often associated with hypomethylation of the *D4Z4* region. NGS analysis revealed the presence of a heterozygous variant (c.5150_5151del) in the exon 41 of *SMCHD1* in the proband, her mother and the uncle. The variant can be classified as pathogenic considering the bioinformatic data and ACMG recommendations (22). In fact, it may create PTC and, consequently, NMD. Even in the case the protein product would be produced, its activity would be impaired because of the lack of SMC hinge domain. Of course, the effective pathogenicity of this variant need to be confirmed by functional assays. Moreover, it is important to remark that affected patients showed reduced methylation levels at the DR1 region and at the region distal to *D4Z4* region (PAS region). This is consistent with the genetic profiles observed in the proband, mother and maternal uncle. Thus, the severe phenotype observed in the proband is explained by the co-occurrence of both genetic features (short fragment, 4qA allele and *SMCHD1* variant), proving that *SMCHD1* genotyping can be helpful to facilitate the diagnosis of FSHD. This case outlines the importance of genetic counseling and familial genetic investigation in the large majority of FSHD cases characterized by inaccurate genotype-phenotype correlation. The reduced penetrance of short alleles at *D4Z4* locus together with a likely underestimation of the variant rate at FSHD2 genes complicate the genetic diagnosis of the disease, the segregation analysis and the calculation of the recurrence risk of FSHD. In this perspective, the analysis of FSHD2 genes should be promoted in a larger number of patients, even in presence of *D4Z4* contractions, to facilitate the genotype-phenotype correlation as well as to provide a more accurate diagnosis and prognosis of disease. To this purpose, prominent attention should be given to the pedigree analysis during the genetic counseling as well as to the segregation analysis among family members of affected patients.

## Ethics statement

This study was carried out in accordance with the recommendations of Santa Lucia Foundation Ethics Committee with written informed consent from all subjects. All subjects gave written informed consent in accordance with the Declaration of Helsinki. The protocol was approved by Santa Lucia Foundation Ethics Committee.

## Author contributions

RC, CS, VC, RG, VE, LC, SZ, and GD made contributions to acquisition of data, analysis, and interpretation of data. GM and MA have been involved in the acquisition of data. RC, CS, GD, LP, and EG have been involved in drafting the manuscript. MS, RP, and LP have been involved in the acquisition of clinical data. RC, CS, SZ, ER, GD, LP, and EG have given final approval of the version to be published.

### Conflict of interest statement

The authors declare that the research was conducted in the absence of any commercial or financial relationships that could be construed as a potential conflict of interest.
